# Incidence and risk factors of postoperative nausea and vomiting following laparoscopic sleeve gastrectomy and its relationship with Helicobacter pylori: A propensity score matching analysis

**DOI:** 10.3389/fendo.2023.1102017

**Published:** 2023-02-22

**Authors:** Yali Song, Jie Zhu, Zhiyong Dong, Cunchuan Wang, Jia Xiao, Wah Yang

**Affiliations:** ^1^Department of Metabolic and Bariatric Surgery, Clinical Research Institute, The First Affiliated Hospital, Jinan University, Guangzhou, China; ^2^Institute of Obesity and Metabolic Disorders, Jinan University, Guangzhou, China; ^3^Laboratory of Metabolic and Molecular Medicine, Guangdong-Hong Kong-Macao Joint University, Guangzhou, China

**Keywords:** nausea, vomiting, sleeve gastrectomy, bariatric surgery, pain, Helicobacter pylori

## Abstract

**Background:**

Postoperative nausea and vomiting (PONV) are common after laparoscopic sleeve gastrectomy (LSG), affecting patient satisfaction and postoperative recovery. The purpose of this study was to investigate the incidence and severity of PONV after LSG and the relationship between Helicobacter pylori (HP) and PONV.

**Methods:**

Patients undergoing LSG in our center from June 1, 2018, to May 31, 2022, were divided into HP-positive and HP-negative groups for retrospective analysis. The independent risk factors of PONV were determined by univariate and binary logistic regression analysis using a 1:1 propensity score matching (PSM) method.

**Results:**

A total of 656 patients was enrolled, and 193 pairs of HP-positive and negative groups were matched after PSM. Both groups of patients had similar clinical features and surgical procedures. PONV occurred in 232 patients (60.1%) after LSG, and the incidence of PONV in HP-positive patients was 61.10%. The incidence and severity of PONV were statistically similar in both groups (P=0.815). Multivariate analysis showed that the female sex (OR=1.644, P=0.042), postoperative pain (OR=2.203, P=0.001) and use of postoperative opioid (OR=2.229, P=0.000) were independent risk factors for PONV after LSG, whereas T2DM (OR=0.510, P=0.009) and OSAS (OR=0.545, P=0.008) independently reduced the incidence rate of PONV. There was no difference either in smoking (P=0.255) or alcohol drinking (P=0.801). HP infection did not affect PONV (P=0.678).

**Conclusions:**

The incidence of PONV following LSG was relatively high. Female sex, postoperative pain and use of postoperative opioid predicted a higher incidence of PONV. Patients with T2DM and OSAS were less likely to have PONV. There was no clear association between HP infection and PONV after LSG.

## Introduction

Overweight and obesity are defined as an excess of body fat accumulation that threatens health. According to the updated data from the World Health Organization, in 2016, more than 1.9 billion adults were overweight globally. Of these, over 650 million were in obesity (1, 2). According to epidemiological studies, obesity can progressively cause and/or exacerbate a wide spectrum of chronic diseases, which include type 2 diabetes mellitus, chronic kidney disease (3), cardiovascular disease (3, 4), a range of musculoskeletal disorders (5, 6), and even certain types of cancer (7). Bariatric surgery becomes necessary for people with severe obesity who cannot sustain weight loss by non-surgical means (e.g., diet and exercise). Laparoscopic sleeve gastrectomy (LSG) has become the most common bariatric surgery because of its simple operation, fewer complications, and good effect in reducing weight and alleviating obesity metabolism-related complications (8–11). Of note, there are a variety of side effects and post-op risks related to bariatric surgery, including acid reflux, dilation of the esophagus, obstruction of the stomach, weight gain or failure to lose weight, infection, and postoperative nausea and vomiting (PONV) (12).

PONV, defined as nausea, vomiting, or retching occurring within 24 h following anesthesia, is the most common adverse reaction after LSG. Without preventive antiemetic treatment, its incidence can reach 80% (13). PONV will induce postoperative discomforts and cause serious complications, such as water-electrolyte disorder and aspiration pneumonia, resulting in prolonged hospitalization and increased medical expenses (14). Previous studies have identified the factors affecting the incidence of PONV came from three aspects: patient factors (e.g., female sex, anxiety, infection, metabolic disease, and gastrointestinal disease), medication/anesthesia factors (e.g., opioids, volatile agents, and nitrous oxide), and surgery factors (e.g., surgical time, procedure, and technique) (15, 16). In adults, the known risk factors for PONV include female sex, non-smoking status, use of postoperative opioids, younger age, and history of PONV or motion sickness (17). However, for obese patients, several factors may contribute to the high susceptibility to PONV. Because patients who undergo bariatric surgery are usually younger women and non-smokers, with laparoscopic or robotic surgery lasting more than one hour, and receive perioperative opioid analgesia, all these are risk factors for PONV. Besides, impaired splanchnic perfusion during pneumoperitoneum and gastric volume reduction (especially after LSG) may further lead to PONV (18–20).

Studies have shown that Helicobacter pylori (HP) infection is closely related to digestive tract diseases such as peptic ulcer, gastric cancer, gastric lymphoma, and chronic gastritis (21). There are many studies on the mechanism, prevention, and treatment, but few on the relationship between HP infection and gastrointestinal adverse reactions such as PONV. Several researches have shown that there is an association between HP and hyperemesis gravidarum, which indicates that HP can exacerbate nausea and vomiting during pregnancy (22–25). Thus, the aims of this retrospective study were to investigate the incidence and risk factors of PONV after LSG and to explore whether HP infection affects PONV in subjects receiving LSG using a propensity score matching (PSM) analysis.

## Methods

### Study population

This study was conducted at the Department of Metabolic and Bariatric Surgery in the First Affiliated Hospital of Jinan University. A preliminary assessment determined surgical qualifications by a multidisciplinary team including surgeons, endocrinologists, anesthesiologists, nutritionists, and nurses. This retrospective study included all patients with obesity who underwent LSG at our bariatric surgery center from June 1, 2018, to May 31, 2022. The exclusion criteria were: (1) age less than 18 years, (2) patients did not undergo HP examination before the operation, (3) patients who were transferred to the intensive care unit (ICU) immediately after the operation, (4) the revision surgery (a repeated surgery due to complications or unsatisfactory results after initial bariatric surgery), (5) patients received HP eradication treatment before the operation, (6) patients received antibiotic treatment within four weeks before the operation, (7) nausea or vomiting before anesthesia.

All Bariatric surgeries were performed by the same well-experienced surgical team. The surgical techniques of LSG and postoperative management were introduced previously (26). On the basis of PONV prophylaxis guidelines, we routinely gave palonosetron and dexamethasone at the end of the operation (13, 27). After surgery, we transferred the patients to post-anesthesia care unit (PACU) until complete recovery and monitored vital signs according to standard clinical practice. In the ward, we used a visual analogue scale (VAS) to evaluate nausea and vomiting or pain (least: 0–10: worst). Depending on the severity of PONV, we decided whether to use antiemetics. For the patients with PONV or cases were intolerable, we usually offered rescue antiemetic agent (including: 5 mg tropisetron, 10 mg metoclopramide or 4 mg ondansetron). On the basis of the level of pain, subjects with postoperative pain received analgesic management, such as flurbiprofen 50 mg, parecoxib 40 mg or tramadol 100 mg (26).

Since (1) we had informed all participants receiving LSG that the clinical data which were acquired during the perioperative period may be retrospectively analyzed and published; And (2) in our study, all data were collected as a regular part of surgical care, and none were designed to collect data specifically for the research, so there was no need for written informed consent. This study protocol was approved by the Ethical Committee of the First Affiliated Hospital of Jinan University (no. KY-2021-070).

### Anesthesia protocol

All procedures were finished under general anesthesia following a standardized clinical routine. Routine monitoring of electrocardiogram, blood pressure, and pulse oximetry were carried out. General anesthesia was induced with propofol, remifentanil, and rocuronium, and the dosage of drugs depended on the body weight of the patient. The maintenance of anesthesia was implemented by the use of remifentanil and propofol, oxygen, and air (26). In accordance with the PONV prevention guidelines, we routinely provided dexamethasone and palonosetron at the end of surgery (13, 27).

### Study outcomes

Nausea is defined as an unpleasant feeling associated with the urge to vomit. Vomiting is defined as successful or unsuccessful (retching) excretion of gastric contents (28). The risk factors and predictors for postoperative nausea and vomiting are generally considered to be almost identical (29). Consequently, nausea or vomiting is not considered as a separate outcome in our research (30). We focused our study on 6 h and 24 h after surgery.

In this study, the primary endpoint was the overall incidence of PONV within 24 h after surgery, with secondary outcomes being the severity of PONV, the type and use of rescue antiemetics, and the time for the first rescue antiemetic and analgesics. Based on the total VAS scores at 6 h and 24 h after operation and the use of rescue antiemetics, two groups were divided (PONV: total VAS score greater than 2 or use of rescue antiemetics; No PONV: total VAS score less than or equal to 2 and no use of rescue antiemetics). Depending on the total postoperative pain VAS (P-VAS) scores at 6 h and 24 h after surgery and the application of rescue analgesics, the definition of postoperative pain was the sum of P-VAS, which was higher than 2 points or applying rescue analgesics. At the same time, for further study, we respectively divided the PONV group and the pain group into three groups: mild (3-6 scores), moderate (7-12 scores) and severe (13-20 scores) (26).

### Data collection

A professional researcher reviewed patients’ electronic medical records and extracted the following data which contained demographic data and perioperative factors. The demographic variables included age, BMI, obesity-related comorbidity [type 2 diabetes mellitus (T2DM), hyperlipidemia (HLP), hypertension], and smoking status. Operational details were collected, mainly including duration of surgery, the use of prophylactic antiemetics and anesthesia methods. We used the C_13_ breath test to detect HP infection.

In our department, the same team performed one standardized questionnaire to all patients. By this way, we acquired the information including PONV score, pain level, alcohol consumption, and smoking status. PONV severity was assessed using the total VAS scores at 6h and 24h after the operation. A higher score indicated more severe nausea and vomiting (31). Pain status was scored with a VAS at 6h and 24h post-operation (32). The alcohol consumption level was quantified before operation using the Alcohol Use Disorders Identification Test (AUDIT) recommended by the World Health Organization. The AUDIT score could be classified into four risk levels: 0 point as a non-drinker; 1-7 points as low risk, 8-15 points as a moderate risk; 16-19 points as high risk; 20 and above as alcohol dependence (33). Smoking status was expressed by the Brinkman index (BI), which is the number of years of smoking multiplied by the number of cigarettes smoked per day. BI results could be divided into four sequential groups: non-smokers as 0; mild smokers as 1-200; moderate smokers as 200-400; and heavy smokers as > 400 (34).

### Statistical analysis

To help overcome the selection bias from the confounding variables, we performed a PSM analysis in each group. The propensity score was calculated by logistic regression analysis. We applied the nearest-neighbor method to match the patients in a 1:1 ratio. As a result, A patient in the HP-positive group was matched with one patient in the HP-negative group. The caliper size was set 0.02 and bad matches were excluded from analysis.

Continuous variables of normal distribution were presented as means ± standard deviations (SD) and were analyzed using an independent t-test. Variables with a skewed distribution were presented as median (interquartile range) and were compared using the Mann-Whitney U-test. Categorical data were presented as percentages and compared using the χ^2^ and Wilcoxon test. The risk factors of PONV post LSG were firstly analyzed by a univariate analysis. After screening the variables, the likelihood ratio stepwise forward method included the significantly related variables in the binary logistic regression analysis. The analysis indexes included the odds ratio (OR), 95% confidence interval (95% CI), and significance test results (P value).

All data were analyzed using SPSS 26.0 software (SPSS Inc., Chicago, IL). All *P* values were two-sided, and *P* < 0.05 was considered statistically significant.

## Results

### Patient characteristics

**Figure 1:**
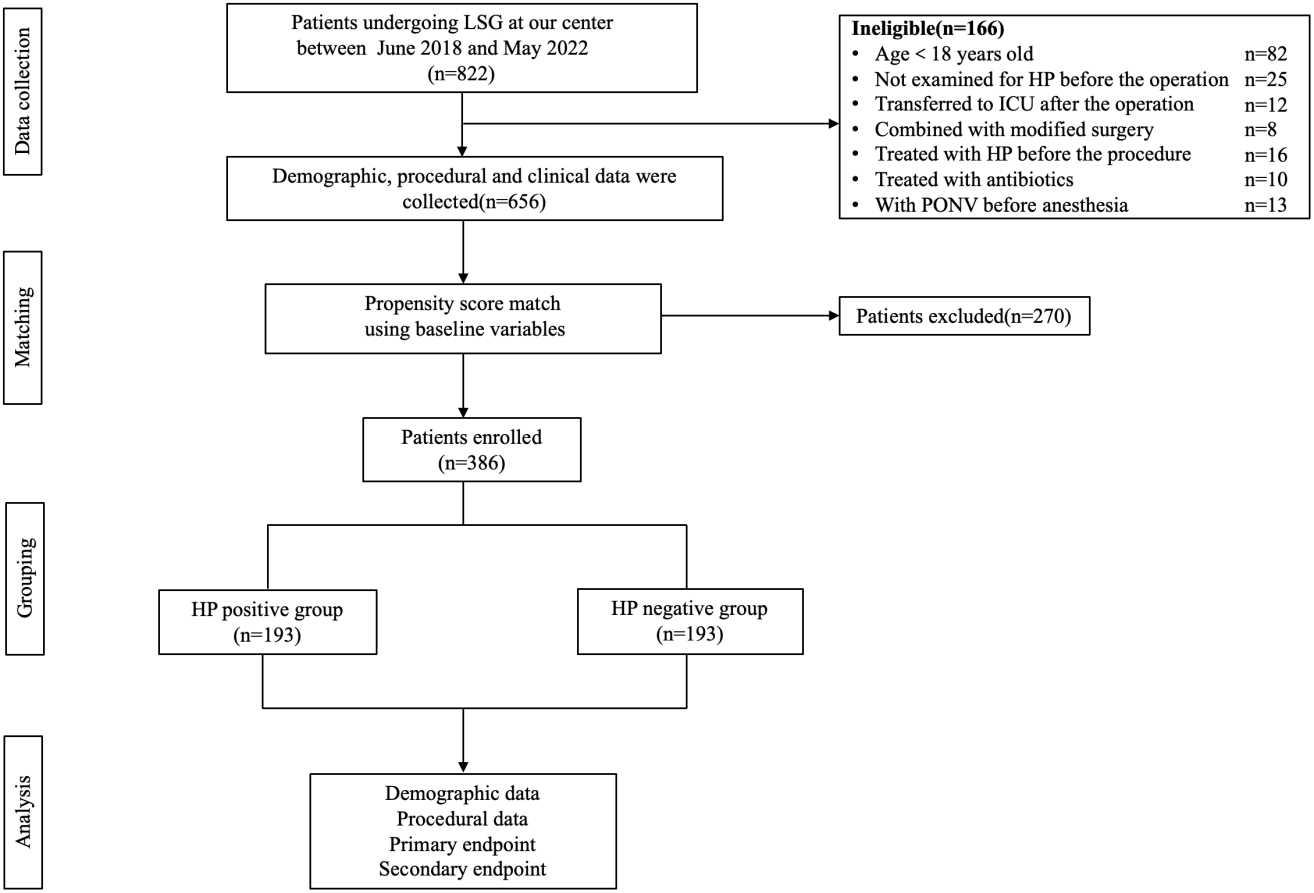
Flow diagram of this study.

The study reviewed 822 patients (205 males and 617 females) who underwent LSG surgery in our hospital between June 1, 2018, and May 31, 2022. In those patients, 82 were younger than 18 years old, 25 were not examined for HP before the operation, 12 cases were transferred to ICU after the operation, 8 patients received revision surgery, 16 cases were treated for HP before the procedure, 10 cases were treated with antibiotics within four weeks before the operation, and 13 cases had nausea and vomiting before anesthesia. Finally, 656 patients were eligible to enter the study prior to the PSM, and we had 193 matched patients over 1:1 PSM, effectively balancing the preoperative confounding factors of the two groups. The research flow chart was shown in **Figure 1**. Demographic data and perioperative factors of all patients before and after PSM were shown in **Table 1**.

**Table 1:** Demographical characteristics and clinical data of the patients (before and after PSM).

Variables	Before PSM			After PSM		
	HP-negative (n=457)	HP-positive (n=199)	P value	HP-negative (n=193)	HP-positive (n=193)	P value
Mean age (years)	30.87 ± 8.28	32.40 ± 7.69	**0.027**	30.70 ± 8.21	32.28 ± 7.68	0.052
Preoperative BMI (kg/m2)	37.28 ± 5.88	37.32 ± 5.49	0.937	37.61 ± 6.34	37.36 ± 5.51	0.683
Postoperative hospital stay (day)	4.04 ± 1.15	3.93 ± 0.94	0.237	4.08 ± 1.08	3.93 ± 0.95	0.146
Operation time (min)	130.65 ± 44.68	132.21 ± 47.56	0.687	131.22 ± 46.57	132.90 ± 47.96	0.728
Blood loss (mL)	13.52 ± 6.24	12.94 ± 6.42	0.276	12.28 ± 3.19	12.03 ± 6.38	0.384
Distance from incisal margin to pylorus (cm)	2.81 ± 0.39	2.76 ± 0.43	0.136	2.77 ± 0.42	2.78 ± 0.42	0.903
Female, n (%)	340 (74.4%)	151 (75.9%)	0.688	140 (72.5%)	146 (75.6%)	0.486
T2DM, n (%)	84 (18.4%)	49 (24.6%)	0.068	38 (19.7%)	45 (23.3%)	0.386
Hypertension, n (%)	94 (20.6%)	41 (20.6%)	0.992	33 (17.1%)	37 (19.2%)	0.597
Hyperlipidemia, n (%)	214 (46.8%)	97 (48.7%)	0.651	81 (42.0%)	95 (49.2%)	0.153
Hyperuricemia, n (%)	286 (62.6%)	105 (52.8%)	**0.018**	98 (50.8%)	105 (54.4%)	0.476
OSAS, n (%)	309 (67.6%)	137 (68.8%)	0.756	128 (66.3%)	131 (67.9%)	0.745
Esophagitis, n (%)	88 (19.3%)	30 (15.1%)	0.200	33 (17.1%)	30 (15.5%)	0.679
Alcohol consumption, n (%)	101 (22.1%)	53 (26.6%)	0.208	60 (31.1%)	50 (25.9%)	0.260
Smoking, n (%)	88 (19.3%)	36 (18.1%)	0.726	41 (21.2%)	35 (18.1%)	0.442
Postoperative pain, n (%)	213 (46.6%)	99 (49.7%)	0.459	88 (45.6%)	95 (49.2%)	0.476

Data are presented as Mean (M) ± Standard Deviation (SD), percentages (%) or median and interquartile range (IQR). Bold is used to highlight statistically significant p-values. PSM, propensity score matching; n, numbers; HP, helicobacter pylori; BMI, body mass index; T2DM, type 2 diabetes mellitus; OSAS, obstructive sleep apnea syndrome.

### Occurrence and severity of PONV in HP-positive group and HP-negative group

Before PSM, there were 390 patients of PONV in 656 patients undergoing LSG, and the infection rate was 59.45%. There was no significant difference in the incidence of PONV between HP-positive and HP-negative patients (P=0.641) (**Table 2**).

**Table 2:** Occurrence of PONV in 656 LSG patients with HP-positive and HP-negative.

	PONV group (n=390)	NoPONV group (n=266)	P value
HP-negative (n=457)	269 (58.9%)	188 (41.1%)	0.641
HP-positive (n=199)	121 (60.8%)	78 (39.2%)	

PONV, postoperative nausea and vomiting; LSG, laparoscopic sleeve gastrectomy; n, numbers; HP, helicobacter pylori.

### Comparison of covariates before and after PSM in groups

Before PSM, there were 199 cases in the HP-positive group and 457 cases in the HP-negative group, respectively. There were significant differences between the two groups in terms of age (P=0.027) and hyperuricemia (P=0.018); After PSM, the infection of HP was taken as the dependent variable, and the above covariates were taken as the independent variables. After 1:1 matching of the data between the two groups, there were 193 cases in each of the two groups. The distribution of the above covariates between the groups reached equilibrium (all P > 0.05) (**Table 1**).

### Comparison of occurrence and severity of PONV

Among the 193 patients in the HP-negative and HP-positive groups, 114 (59.1%) and 118 (61.1%) developed PONV within 24 h after the operation. Most PONV cases were mild. The incidence, severity (P=0.851), frequency of rescue antiemetics (P=0.615), and the earliest antiemetics use (P=0.359) in the two groups were not statistically significant (**Table 3**).

**Table 3:** Occurrence and severity of PONV and the Use of rescue antiemetics (n=193).

Variables	HP-negative (n=193)	HP-positive (n=193)		P value	
*Severity of PONV*			Z=0.188	0.851
NO	79 (40.9%)	75 (38.9%)			
Mild	74 (38.4%)	87 (45.1%)			
Moderate	32 (16.6%)	24 (12.4%)			
Severe	8 (4.1%)	7 (3.6%)			
*Times of rescue antiemetics*			Z=0.503	0.615
NO	121 (62.7%)	113 (58.5%)			
1 time	38 (19.7%)	50 (25.9%)			
2 times	22 (11.4%)	20 (10.4%)			
≥ 3 times	12 (6.2%)	10 (5.2%)			
*Earliest of having antiemetics*			Z=0.918	0.359
No	106 (54.9%)	100 (51.8%)			
0-6 h after surgery	21 (10.9%)	18 (9.3%)			
6-12 h after surgery	30 (15.5%)	27 (14.0%)			
12-24 h after surgery	22 (11.4%)	34 (17.6%)			
> 24 h	14 (7.3%)	14 (7.3%)			

Data are presented as percentages (%). PONV, postoperative nausea and vomiting; HP, helicobacter pylori.

### Univariate analysis

After PSM, 386 patients were finally included, including 100 males and 286 females. A total of 232 occurred PONV, with an incidence rate of 60.1%. According to PONV occurrence, those patients were divided into the PONV group and the no PONV group. The univariate analysis showed that females had a significantly higher risk of PONV than males after LSG (P=0.008). The incidence rate of PONV in patients with diabetes (P=0.003) and OSAS was lower than in those who had not those complications (P=0.007). The incidence of PONV was significantly higher in patients with postoperative pain (P=0.000) and use of postoperative opioid (P=0.001) than in patients without pain (**Table 4**).

**Table 4:** Univariate analysis of PONV after LSG (after PSM).

Variables	PONV Group (n=232)	NoPONV Group (n=154)	P value
Mean age (years)	31.41 ± 8.10	31.62 ± 7.83	0.803
Preoperative BMI (kg/m^2^)	37.03 ± 5.76	38.18 ± 6.14	0.062
postoperative hospital stay (day)	4.16 ± 1.20	4.12 ± 0.97	0.727
Operation time (min)	128.67 ± 40.10	137.17 ± 56.02	0.105
Blood loss (mL)	11.68 ± 5.20	11.62 ± 5.27	0.916
Distance from incisal margin to pylorus (cm)	2.78 ± 0.42	2.77 ± 0.43	0.749
Gender, n(%)	183 (78.9%)	103 (66.9%)	**0.008**
T2DM, n(%)	38 (16.4%)	45 (29.2%)	**0.003**
Hypertension, n(%)	38 (16.4%)	21 (13.6%)	0.463
Hyperlipidemia, n(%)	105 (45.3%)	71 (46.1%)	0.870
Hyperuricemia, n(%)	117 (50.4%)	86 (55.8%)	0.297
OSAS, n(%)	128 (55.2%)	106 (68.8%)	**0.007**
Esophagitis, n(%)	34 (14.7%)	29 (18.8%)	0.277
HP-positive, n(%)	118 (50.9%)	75 (48.7%)	0.678
*Alcohol consumption, n(%)*			0.801
Non-drinker	166 (71.6%)	110 (71.4%)	–
Low-risk	26 (11.2%)	12 (7.8%)	–
Moderate-risk	13 (5.6%)	10 (6.5%)	–
High-risk	7 (3.0%)	5 (3.3%)	–
Alcohol dependence	20 (8.6%)	17 (11.0%)	–
*Smoking habit, n(%)*			0.255
Non-smoker	182 (78.4%)	128 (83.1%)	–
Light smoker	38 (16.4%)	20 (13.0%)	–
Moderate smoker	6 (2.6%)	4 (2.6%)	–
Heavy smoker	6 (2.6%)	2 (1.3%)	–
*Postoperative pain, n(%)*			**0.000**
No	105 (45.3%)	98 (63.6%)	–
Mild	95 (40.9%)	46 (29.9%)	–
Moderate	25 (10.8%)	8 (5.2%)	–
Severe	7 (3.0%)	2 (1.3%)	–
*Use of postoperative opioid, n(%)*			**0.001**
NO	105 (45.3%)	98 (63.6%)	-
1 time	90 (38.7%)	40 (26.0%)	-
2 times	25 (10.8%)	14 (9.1%)	-
≥ 3 times	12 (5.2%)	2 (1.3%)	-

Data are presented as Mean (M) ± Standard Deviation (SD), percentages (%) or median and interquartile range (IQR). Bold is used to highlight statistically significant p-values. PONV, postoperative nausea and vomiting; LSG, laparoscopic sleeve gastrectomy; PSM, propensity score matching; BMI, body mass index; T2DM, type 2 diabetes mellitus; OSAS, obstructive sleep apnea syndrome; HP, helicobacter pylori.

### Multivariate regression analysis

Significant and independent predictors of PONV incidence were determined by a multivariate logistic regression analysis. Variables that were statistically significant in the univariate analysis were included in the multivariate logistic regression analysis model. Results showed that female sex and postoperative pain were important independent predictors of the increase in the incidence rate of PONV. At the same time, type 2 diabetes (T2DM) and OSAS significantly and independently reduced the incidence rate of PONV after adjusting for confounding variables. The OR (95% CIs, P value) of PONV incidence after LSG was 1.644 (1.017-2.655, P = 0.042) in females and 2.203 (1.430-3.396, P = 0.001) in the pain group; The group of use of postoperative opioid was 2.229 (1.446-3.434, P = 0.000); T2DM group was 0.510 (0.306-0.848, P = 0.009) and OSAS group was 0.545 (0.349-0.853, P = 0.008) (**Table 5**).

**Table 5:** Odds ratio (OR) and 95% confidence intervals (CI) of risk factors for PONV after LSG (after PSM).

	OR (95%CI)	P value
Female gender	1.644 (1.017-2.655)	**0.042**
T2DM	0.510 (0.306-0.848)	**0.009**
OSAS	0.545 (0.349-0.853)	**0.008**
Postoperative pain	2.203 (1.430-3.396)	**0.001**
Use of postoperative opioid	2.229 (1.446-3.434)	**0.000**

Bold is used to highlight statistically significant p-values. OR, odds ratio; CI, confidence intervals; T2DM, type 2 diabetes mellitus; OSAS, obstructive sleep apnea syndrome.

## Discussion

### Incidence and severity of PONV

Previous studies have shown that postoperative PONV was the most common adverse effect of weight loss surgery, and its overall incidence exceeded 80% in some types of surgery (35). PONV following LSG was thought to be secondary to the sharp reduction of gastric volume and increased intragastric pressure (36). A retrospective chart review study showed that the incidence of PONV in the LSG group (66.9%) was higher than that in the primary laparoscopic Roux-en-Y gastric bypass group (33.1%) (37). Another study pointed out that 65% of patients experience PONV within the first 24 h following LSG (18). Our study found that the incidence of PONV in Chinese patients at 0-24 h following LSG was 60.1%, lower than the above reported incidences of patients from other countries (38–40). This could be attributed to: in our center, tropisetron hydrochloride (a potent and selective 5-HT3 receptor antagonist) and metoclopramide (a dopamine antagonist) were routinely used during and right after the surgery to prevent PONV (41, 42).

### Biological sex and PONV

It was identified that the female sex predicted a higher incidence of PONV following surgery (43–45). Halliday et al. Found that when two or even three preventive drugs were used, the incidence of PONV in female patients was still as high as 78% following weight loss surgery, which was three times than that of male patients during the same period (18). Another retrospective study showed that preventive antiemetic therapy did not have an ideal effect on preventing and treating PONV after weight loss surgery. After drug intervention, the incidence of PONV in female patients was still nearly 1/3 higher than that in male patients (60.4% vs. 42.9%), suggesting that the risk of PONV after bariatric surgery in female patients will not be significantly reduced with the use of preventive drugs (19). The incidence rate of PONV varies with the different phases of the menstrual cycle (19, 46, 47). However, this conclusion was contradicted by a randomized controlled trial study involving more than 5,000 patients in 2007, in which no association between the menstrual cycle stage or menopausal status and the incidence of PONV was identified (48). The molecular mechanism responsible for the correlation between the female sex and the incidence rate of PONV is still largely unknown.

### Postoperative pain and PONV

Previous studies had shown that PONV was strongly associated with postoperative pain in LSG (26). Our study also demonstrated that postoperative pain was a risk factor for PONV after LSG. The possible reasons could be (1): in essence, high pain intensity was inclined to increase the risk of PONV, and (2) in our center, opioids, such as tramadol, were preferred for postoperative pain, which may increase the risk of PONV and constituted one of the major risk factors in the scoring system (49, 50). However, further study was warranted to confirm the impact of postoperative pain on PONV after LSG.

### OSAS and PONV

A major finding of our research was that patients without OSAS had a higher risk of PONV than patients with OSAS. Obesity is considered the main factor leading to OSAS, of which severity could be measured by the sleep apnea-hypopnea index (AHI). With the increase in BMI, the AHI of both males and females increases, and this trend is tendentious in males (51). Although OSAS did not affect the prognosis of bariatric surgery, it indeed affected the postoperative complication of cardiopulmonary function (52). Continuous positive airway pressure (CPAP) is currently the most effective method for treating moderate to severe OSAS, which improves the respiratory function of patients with morbid obesity and accelerates the reconstruction of preoperative pulmonary function (53). A previous study found that in subjects receiving Roux-en-Y gastric bypass, the no-CPAP group reported a higher incidence of oxygenation disturbance, but a slightly lower incidence, although not statistically significant, of PONV when compared with the CPAP group (54). Thus, a possible reason for the lower incidence of PONV in patients with OSAS in this study is the routine use of CPAP in the perioperative period of LSG. More substantial evidence and molecular pathway for this conclusion warrant further investigations.

### Alcohol drinking, smoking, and PONV

A recent study reported decreased risks of PONV in alcoholics than non-drinkers and light-drinkers who underwent abdominal surgery (55). In addition, since chronic alcoholics have higher basal activity of cytochrome P450 2E1 (CYP2E1), which also accelerates the metabolic rate of volatile anesthetics, the main reason of PONV within the first two hours after surgery moderate- or heavy-drinkers (including alcohol dependence patients) may expect a reduced incidence of PONV post-LSG (56, 57). This is not consistent with what we demonstrated here. Previous studies have also built an association between the reduced incidence of PONV and cigarette smoking in Bariatric surgeries (58). However, we did not observe such correlations in our study.

### HP and PONV

A previous study has demonstrated no association between HP infection and nausea after general anesthesia (59). Notash et al. Also found no relationship between HP infection and PONV who underwent general and urological surgery (60). The incidence of PONV in our research was similar in HP-positive and negative patients. Although HP may be related to severe pregnancy-related vomiting, it did not exacerbate LSG-related nausea. In bariatric surgery, to our best knowledge, this is the first report showing that HP infection did not affect the prevalence of PONV after LSG. However, since our research is a single center, more extensive cohort studies are needed for the validation of this conclusion.

### Strengths and limitations

There are some limitations in this retrospective study: (1) The confirmation of a PONV event was determined by using rescue antiemetics or notating its manifestation in the medical records. This approach raises the possibility that the PONV frequencies were underestimated as some patients might experience untreated PONV; (2) Other potential factors, such as PONV history, migraine, and duration of anesthesia, were not considered, which may bias the results; (3) Since we only observed the PONV incidence within 24 h post-operation, a long-term follow-up study is needed to confirm and expand the conclusions; (4) Mechanistic research is required to investigate the molecular pathways leading to PONV after LSG and other types of bariatric surgery. However, as far as we know, this is the largest reported sample size in the study of PONV in LSG. Those confounding factors could be counterpoised after PSM. The relationship between HP and PONV in populations undergoing LSG was also interpreted. Further basic research is required to investigate the molecular mechanism leading to PONV after LSG and other types of bariatric surgery.

## Conclusions

In conclusion, the incidence of PONV after LSG is relatively high. Female sex, postoperative pain and use of postoperative opioid predicted a higher incidence of PONV. Patients with T2DM and OSAS had less likelihood of a related PONV. There was no clear association between HP infection and PONV after LSG.

## Data availability statement

The original contributions presented in the study are included in the article/supplementary materials. Further inquiries can be directed to the corresponding authors.

## Ethics statement

As all participants receiving LSG were informed that the clinical data which were acquired during the perioperative period may be retrospectively analyzed and published, and all data were collected as a standard part of surgical care, and none were designed to collect data specifically for the research, written informed consent was not required. This study protocol was approved by the Ethical Committee of the First Affiliated Hospital of Jinan University (no. KY-2021-070).

## Author contributions

YS, JZ, and WY designed the study. YS collected patients’ data. YS, JZ, and JX performed the analyses and wrote the paper. ZD and CW assisted with the study design and analysis. All authors contributed to the article and approved the submitted version.
